# Effect of discontinuing ongoing education and postprescription feedback on antimicrobial prescriptions at discharge from the emergency department

**DOI:** 10.1017/ash.2022.19

**Published:** 2022-07-18

**Authors:** Yasuaki Tagashira

**Affiliations:** 1 Department of Infectious Diseases, Tokyo Medical and Dental University Graduate School of Medical and Dental Sciences, Tokyo Japan; 2 Department of Infectious Diseases, Tokyo Metropolitan Tama Medical Center, Tokyo, Japan

## Abstract

Multifaceted intervention is preferrable as an ASP strategy in the emergency department (ED). I assessed the effect of discontinuing multifaceted intervention for antimicrobial prescriptions at discharge in the emergency department. The proportion of appropriate prescriptions decreased quickly after discontinuation. Continuous commitment to appropriate antimicrobial prescriptions is needed for effective antimicrobial stewardship.

The antimicrobial stewardship program (ASP) is a core strategy for optimizing the use of antimicrobial prescriptions at discharge (APDs) in the emergency department (ED). Multifaceted intervention by infectious disease physicians is effective in promoting appropriate APDs in the ED,^1^ but securing the time and personnel needed to maintain such an intervention, including postprescription review and feedback (PPRF), is challenging.

Discontinuing antimicrobial stewardship in other settings, including the primary care and inpatient settings, reportedly has had various outcomes.^2–4^ The effect of discontinuing core interventions, such as educational sessions and PPRF in the ED, is not well understood. In the present study, I examined changes in the average proportion of monthly APDs after infectious disease physicians discontinued multifaceted intervention in the ED.

## Materials and methods

This quasi-experimental, observational study was conducted at Tokyo Metropolitan Tama Medical Center, a tertiary care center in Tokyo. The study period included a 1-year ASP implementation period (October 2018–September 2019) followed by an 18-month postintervention period (October 2019–March 2021). The multifaceted intervention, aimed at reducing APDs in the ED, was conducted during the implementation of an ASP. Details of the intervention have been described elsewhere.^1^ During the postintervention period, educational sessions, PPRF, and monthly reports from a designated infectious disease (ID) physician were discontinued. Physicians newly recruited after the intervention period were only given a pocket guide on evidence-based treatments, and they were informed about the antimicrobial order sets for common infectious diseases in the electronic medical records (EMRs) during the orientation. All patients visiting the ED during the study period were initially enrolled; among them, patients discharged home with an APD from the ED were extracted for analysis. The exclusion criteria were based on a previous study.^1^

A flow diagram for assessing the necessity and appropriateness of APDs was presented in a previous study.^5^ Antimicrobial misuse was defined as unnecessary (ie, the type of antimicrobial was not indicated), inappropriate (ie, the chosen antimicrobial was considered ineffective or not recommended), or suboptimal (ie, the route, interval or dosage was incorrect) based on the previously mentioned criteria (Supplementary Table 1). All APDs not meeting the classification of misuse were considered appropriate. The primary outcome was the change in the average proportion of monthly APDs per 1,000 visits to the ED. The χ^2^ test or Fisher exact test was used to compare 2-tailed categorical variables, and the Mann-Whitney *U* test was used to compare continuous variables. Segmented regression in interrupted time-series analysis (ITSA) was used to assess changes in slope and changes in intercept of the monthly proportion of all types of APD per 1,000 visits. The requirement for patient consent was waived because the study was an institutional quality improvement project. The institutional review board at Tokyo Metropolitan Tama Medical Center approved the study.

## Results

In total, 78,586 patients visited the ED during the study period; of these, 33,785 visited during the intervention period and 44,801 visited during the postintervention period. However, 104 patients (0.1%) were excluded, leaving 1,280 (3.8%) with an APD during the intervention period and 1,880 (4.2%) patients with an APD during the postintervention period who were finally enrolled for analysis (Supplementary Fig. 1). The median patient age was 52 years (range, 15–104), and 50.9% were female. During the intervention period, 250 physicians prescribed antimicrobials in the ED, and their median postgraduate years (PGY) was 4 years (range, 2–44). During the postintervention period, 285 physicians prescribed antimicrobials in the ED, and their median PGY was 5 years (range, 2–36 years) (Supplementary Table 2). Moreover, 87 physicians (30.1%) were newly recruited after discontinuing the multifaceted intervention and had at least 1 APD in the ED.

Although the average monthly proportion of appropriate APDs was 79.5% in the intervention period and 80.8% in the postintervention period, respectively, ITSA revealed an immediate decrease in the number of appropriate APDs per 1,000 visits (−7.31; 95% confidence interval [CI], −13.0 to −1.6; *P* = .001 for intercept) as well as a decrease in the trend (−1.1; 95% CI, −1.92 to −0.27; *P* = .001 for trend) (Table 1). The immediate change in the average monthly proportion of all types of misuse of APDs increased, although the change was not statistically significant. Moreover, an increasing trend in the average monthly proportion of inappropriate APDs was observed (+0.78; 95% CI, 0.38–1.2; *P* < .001 for trend) (Supplementary Fig. 2). The proportion of appropriate APDs among newly recruited physicians was significantly lower than among physicians who had experienced multifaceted intervention performed by an infectious disease physician during the intervention period (Table 2).

**Table 1.** Interrupted Time-Series Analysis of Changes in APD Trends^a^

Variable	Regression Intercept	Intervention Trend	Change After the Stop of Intervention	*P* Value	Change in Trend During Postintervention Period	*P* Value
No. of APDs	32.82 (28.31 to 37.33)	+0.89 (0.23 to 1.55)	−3.89 (−11.92 to 4.14)	.33	−0.50 (−1.49 to 0.50)	.32
Proportion of appropriate APDs, % (95% CI)	72.92 (66.69 to 79.14)	+1.20 (0.48 to 1.92)	−7.31 (−13.04 to −1.59)	.01	−1.10 (−1.92 to −0.27)	.01
Proportion of overall misuse of APDs, % (95% CI)	27.10 (20.90 to 33.30)	−1.20 (−1.92 to −0.48)	+6.63 (0.87 to 12.39)	.03	+1.11 (0.29 to 1.93)	.01
Unnecessary APDs, % (95% CI)	10.20 (5.29 to 15.11)	−0.25 (−0.85 to 0.35)	+3.61 (−0.56 to 7.78)	.09	+0.17 (−0.48 to 0.83)	.60
Inappropriate APDs, % (95% CI)	12.59 (10.07 to 15.11)	−0.73 (−1.07 to −0.39)	+2.80 (−0.20 to 5.79)	.07	+0.78 (0.38 to 1.17)	<.001
Suboptimal APDs, % (95% CI)	4.33 (2.25 to 6.40)	−0.22 (−0.50 to 0.06)	+0.22 (−1.91 to 2.35)	.83	+0.17 (−1.40 to 0.47)	.28

a Data are presented as mean monthly prescriptions per 1,000 visits with 95% confidence intervals unless otherwise specified.

**Table 2.** Comparison of Physicians Prescribing Discharge Antimicrobials in the Emergency Department After Discontinuation of Intervention

Variable	APDs Prescribed by Physicians During Multifaceted Intervention (N=1,405), No. (%)	APDs Prescribed by Physicians Without Multifaceted Intervention (N=475), No. (%)	*P* Value
Proportion of appropriate APDs	1,173 (83.4)	341 (71.8)	<.001
Proportion of overall misuse of APDs	232 (16.5)	134 (28.2)	<.001
Proportion of unnecessary APDs	136 (9.7)	67 (14.1)	<.001
Proportion of inappropriate APDs	80 (5.7)	52 (10.9)	.0002
Proportion of suboptimal APDs	16 (1.1)	15 (3.2)	.006

## Discussion

The proportion of appropriate APDs between the intervention and discontinuation periods in the ED demonstrated a decreasing tendency immediately after the intervention was discontinued. Moreover, the proportion of inappropriate APDs increased, suggesting that providing education and PPRF to individual physicians is vital to promoting and maintaining appropriate APDs in the ED.

The impact of discontinuing the intervention was felt immediately in the primary care and inpatient settings,^3,4^ suggesting that the same may hold true in the ED, in line with previous reports.^6^ A possible reason for the increase in the proportion of inappropriate APDs may be a decline in the use of the evidenced-based treatment pocket guide and order sets in the EMRs (the guide and order sets). Thus, it may be important to provide immediate PPRF and encourage using the guide and order sets through feedback. On the other hand, considering only the postintervention trend, the proportion of appropriate APDs apparently remained stable even amid the COVID-19 pandemic (0.10; 95% CI, −0.27 to 0.47; *P* = .55 for postintervention trend). The guide and order sets might have contributed to this finding. Moreover, the discontinuation of ongoing education and PPRF revealed that the guide and order sets helped to standardize the route, dosage, and intervals of APDs. On the other hand, the guide and order sets did not sufficiently compensate for the loss of knowledge previously provided by PPRF. This finding suggests that each intervention may impact different categories of misuse of APDs and is also interactional in appropriate APDs by physicians.

During the study period, ∼30% of the physicians were newly hired, prescribed APDs after the intervention was discontinued, and prescribed 475 (25%) of all 1,880 APDs. Physician turnover and the misuse of APDs by newly hired physicians were obstacles to improving and maintaining appropriate APDs. Pregraduate education, education during residency, and education and intervention for appropriate antimicrobial use beyond the confines of a single hospital may be a necessary framework for ASP in battling antimicrobial resistance. The requirement for antimicrobial stewardship teams has increased over the years.^7^ With the increasing need for an ASP in various settings, more efficient methods of promoting the appropriate use of antimicrobial agents under the conditions of time and personnel limitations are desirable.

This study had several limitations. As a monocentric study, the findings may not be generalizable to other institutions. Patients’ comorbidity and severity of illness per APD were unable to be tracked or evaluated. The appropriateness of individual physicians’ APDs was not monitored, and individual differences in oral antimicrobial prescribing behavior were not examined. Assessing the appropriateness of individual physician prescriptions will doubtlessly lead to more effective and efficient antimicrobial stewardship. No assessment has yet been made of the impact of reducing interventions on ID physicians’ time or on decreasing medical costs. Finally, the impact of the national action plan for antimicrobial resistance, pregraduate education, and the previous hospital’s ASP on newly recruited physicians was not assessed.

In conclusion, continuous education and intervention by ID specialists are important for improving and maintaining appropriate APDs in the ED. As the number of settings in which ASPs are implemented increases, providing the requisite education to newly hired physicians and continuously conducting PPRF under the conditions of limited time and personnel will become more urgent.

## Supplementary material

For supplementary material accompanying this paper visit https://doi.org/10.1017/ash.2022.19. click here to view supplementary material
