# Supplementary material for: Effect of discontinuing ongoing education and postprescription feedback on antimicrobial prescriptions at discharge from the emergency department
Source: Antimicrob Steward Healthc Epidemiol. 2022 Jul 18;2(1):e124. doi: 10.1017/ash.2022.19 (PMC9726547; doi:10.1017/ash.2022.19)
Supplement: Supplementary file 1 [file S2732494X22000195sup001.docx]

Supplementary: Effect of discontinuing ongoing education and post-prescription feedback on antimicrobial prescriptions at discharge from the emergency department

**Authors:** Yasuaki Tagashira, MD, PhD

Supplementary Table 1. The definition and example of all categories of misuse of APDs

| Category | The definition | Example |
| --- | --- | --- |
| Unnecessary | Unnecessary use was defined as the use of an antimicrobial agent for patients with no indications, procedures with no indications, a non-infectious condition, nonbacterial infection or self-limiting bacterial infection and included antimicrobial use in the context of an uncertain diagnosis. | Antimicrobials for viral upper respiratory tract infections.  Treatment of gastroenteritis outside established indications. |
| Inappropriate | Inappropriate use was defined as the use of an antimicrobial agent not conforming to the current prophylaxis or treatment guidelines, including agents with a too broad or too narrow spectrum. | Use of levofloxacin to treat cellulitis despite absence of antimicrobial allergy. |
| Suboptimal | Suboptimal use was defined as the use of antimicrobials for established infections that can be improved via changing the drug route, drug interval or drug dose. | Failure to adjust doses of renally cleared drugs. |

Supplementary Table 2. Characteristics of physicians prescribing discharge antimicrobials in the emergency department

| Characteristics | Intervention period  (N=1,280) | Post-intervention period  (N=1,880) |
| --- | --- | --- |
| Department |  |  |
| Emergency Department | 832 (65.0) | 1184 (65.0) |
| Department of Medicine ^a^ | 128 (10.0) | 191 (10.0) |
| Department of Surgery ^b^ | 320 (25.0) | 505 (25.0) |
| Occupational status of prescribing physicians |  |  |
| Resident | 832 (65.0) | 1184 (63.0) |
| Physician in a medical subspecialty ^c^ | 128 (10.0) | 191 (10.2) |
| Physician in a surgical subspecialty ^c^ | 320 (25.0) | 505 (26.9) |
| Prescribing physician’s post graduate year |  |  |
| ≤ 3 | 459 (35.9) | 688 (36.6) |
| 4-7 | 561 (43.8) | 796 (42.3) |
| ≥ 8 | 260 (20.3) | 396 (21.1) |
| Sex |  |  |
| Male | 840 (65.6) | 1137 (60.5) |
| Female | 440 (34.4) | 743 (39.5) |

NOTE.

Data are presented as a number (%) unless otherwise specified.

Abbreviations: ED, emergency department

a Medical department includes the general medicine, pulmonary, gastroenterology, nephrology, and infectious diseases departments.

b Surgical department includes the general surgery, otorhinolaryngology, urology,

obstetrics/gynecology, oral surgery, orthopedics, plastic surgery, neurosurgery, and dermatology departments.

c Physicians in subspecialties include subspecialty fellows and attending physicians.

Supplementary Figure 1. Description of the study population


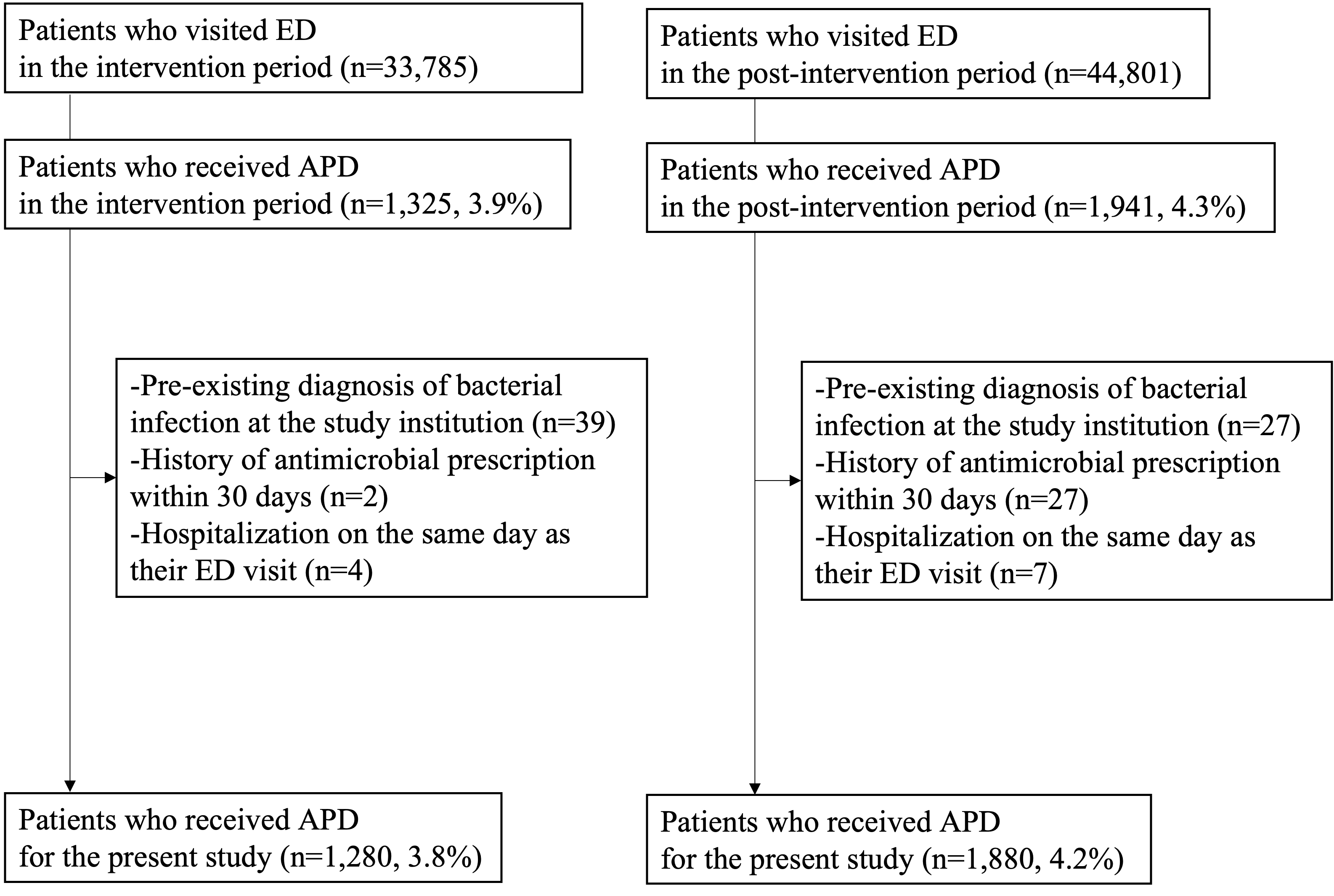


Supplementary Figure 2. Changes in the proportion of appropriate APDs and all types of misused APD per 1,000 visits to the ED
